# Hybrid Helmholtz–Helical Metamaterial for Broadband-Targeted Suppression of Substation Noise

**DOI:** 10.3390/ma19030579

**Published:** 2026-02-02

**Authors:** Jingkai Nie, Yi Tian, Xing Li, Qiang He, Weichun Huang, Yu Han, Xiaogang Chen, Ming-Hui Lu

**Affiliations:** 1 China Electric Power Research Institute Co., Ltd., Beijing 102211, China; niejingkai1@epri.sgcc.com.cn (J.N.); heqiang0927@163.com (Q.H.); hanyu1@epri.sgcc.com.cn (Y.H.); 2 National Laboratory of Solid State Microstructures & Collaborative Innovation Center of Advanced Microstructures, Nanjing University, Nanjing 210093, China; weichunhuang@nju.edu.cn; 3 Department of Materials Science and Engineering, Nanjing University, Nanjing 210093, China; 4 State Grid Zhejiang Electric Power Co., Ltd., Hangzhou 310063, China; carlcxg1980@126.com; 5 International Institute of Acoustic Technology, Suzhou 215513, China

**Keywords:** urban substations, low-frequency noise, Helmholtz resonator, acoustic metamaterial

## Abstract

Low-frequency noise, primarily generated by transformers and electrical machinery in substations, presents considerable environmental and health risks due to its strong penetration and minimal attenuation. Conventional noise control methods often fail to effectively absorb such low-frequency sounds. In response to this challenge, acoustic metamaterials featuring unique subwavelength structures have emerged as a promising solution for absorbing low-frequency and broadband noise. This study introduces a novel sound-absorbing metamaterial that integrates parallel-connected Helmholtz resonators with a helical cavity structure. To enhance its performance across a broad frequency range, the metamaterial is optimized using a genetic algorithm. Experimental validation, based on 3D-printed samples and impedance tube measurements, demonstrates high absorption efficiency at target frequencies (100 Hz, 300 Hz, and 500–1300 Hz), with absorption coefficients exceeding 0.9. The results confirm that the metamaterial effectively reduces low-frequency core noise. This work represents a significant advancement in noise control technologies for substations, with broader implications for urban noise mitigation and environmental protection.

## 1. Introduction

Low-frequency noise, characterized by its strong penetration and minimal attenuation, has emerged as a growing environmental concern. Recognized as one of the major environmental pollutants, it poses severe implications for both human health and the natural environment [1,2]. Substations, essential components of power systems, often generate low-frequency noise, primarily from electrical transformers and other machinery [3,4,5]. Many substations are located in densely populated residential areas, where industrial noise disrupts daily life for local residents. Transformer noise can span a broad frequency range, from 50 Hz to over 1300 Hz, encompassing both tonal and broadband components [6]. To mitigate noise at the most impactful frequencies—specifically 100 Hz and 300 Hz (Table 1)—while also addressing broader frequencies from 500 Hz to 1300 Hz, commonly associated with broadband noise from transformer cooling systems. Combining these frequency targets offers a more comprehensive noise reduction strategy.

Traditional noise reduction methods, including the use of sound-absorbing materials such as fibers, foam, and metal panels, are commonly employed to address this issue [7,8,9]. However, these materials are less effective at absorbing low-frequency noise due to their limited energy dissipation capacity [10,11]. Furthermore, they often require thick layers, comparable to the wavelength of sound waves, which reduces their effectiveness in environments like substations. Therefore, more efficient solutions are urgently needed to control low-frequency noise.

For low-frequency sound absorption, two main approaches are commonly used: the dissipative approach, which utilizes porous materials and their structurally modulated derivatives that rely on viscous-thermal losses within intricate microstructures for broadband performance, and the resonant approach, exemplified by acoustic metamaterials, which achieve targeted, deep subwavelength absorption through engineered local resonances. While porous materials are well-established, acoustic metamaterials have gained particular recognition for their exceptional, tunable performance in low-frequency noise control due to their superior ability to achieve precise impedance matching at sub-wavelength scales.

Acoustic metamaterials have garnered significant attention as a promising solution for sound absorption and noise control. With their unique subwavelength structures, these materials offer the potential for optimal performance in noise mitigation [12,13,14,15,16,17,18]. Acoustic metamaterials are designed to selectively control sound waves by creating band gaps, providing properties such as frequency tuning, negative refraction, and multifunctionality [19,20,21]. Among these, Helmholtz-based acoustic metamaterials are particularly recognized for their excellent performance in absorbing low-frequency noise.

Over the past two decades, various resonant structures—such as Helmholtz resonators (HR) [22,23,24,25], membrane resonators (MR) [26,27,28], micro-perforated panels (MPP) [11,29,30,31], and Fabry-Pérot (FP) resonators [32,33,34]—have emerged as effective solutions for noise control. A notable example is the Jiaye Substation in Guangzhou, China, the first “ultra-silent” substation located in a high-density residential area. This substation has successfully implemented noise control measures, including the use of acoustic insulation doors made from metamaterials, which have significantly reduced low-frequency noise. Despite these advancements [35,36], achieving effective low-frequency and broadband sound absorption remains a significant challenge in noise control research.

In this paper, we aim to address these challenges by introducing a novel sound-absorbing metamaterial that combines parallel-connected Helmholtz resonators with a helical cavity structure. The design is specifically tailored to mitigate the low-frequency noise generated by electrical equipment. The use of a genetic algorithm to optimize the metamaterial’s parameters further enhances its performance in sound absorption. Experimental results confirm the effectiveness of this novel metamaterial in reducing low-frequency noise, with a broad absorption range from 100 to 1000 Hz, offering a promising solution for noise control in substations.

## 2. Acoustic Modeling of Substations

To design acoustic metamaterials that mitigate low-frequency noise generated by power equipment, it is crucial to thoroughly understand the noise sources and their characteristics. Investigations of urban substations indicate that transformers are the primary source of this noise. The noise in transformers is primarily caused by magnetostrictive deformation of the core and periodic deformation of the oil tank [37,38]. Specifically, the core noise results from the periodic magnetostriction of the silicon steel sheets at varying excitation frequencies, as well as from the periodic deformation induced by the electromagnetic attraction between the seams of the sheets and their laminations [39,40]. Several factors contribute to the overall noise, including the vibration of the core, the periodic deformation of the device coils, and the resonance of the device. Moreover, both the core and device vibrations are transmitted through various paths to the exterior of the oil tank, leading to significant noise and vibration from the transformer [41,42]. Based on noise measurements taken from approximately 50 urban substations across Beijing, Zhejiang, Henan, Hebei, and other provinces, this paper identifies the key frequency bands that influence transformer noise, as shown in Table 1. According to the data in Table 1, the design frequencies were selected at 100 Hz and 300 Hz for single-point noise mitigation. Additionally, to ensure broader structural effectiveness, broadband absorption was considered between 500 Hz and 1400 Hz.

Noise measurements from urban substations indicate that certain transformers generate broadband noise when their cooling fans operate. Specifically, noise from transformers includes significant low-frequency noise caused by magnetostriction in the core, as well as broadband noise resulting from the cooling equipment, such as fans and pumps, particularly in forced oil circulation systems for large transformers. These cooling systems contribute additional noise within the mid-to-high frequency range, typically between 1 kHz and 2 kHz. In addition to the noise identified in Table 1, high-frequency noise also requires effective mitigation. To address this, a broadband sound-absorbing material was developed. This material reduces low-frequency noise at specific points and provides broadband absorption for mid- and high-frequency noise. Due to the wide range of noise frequencies and the limited number of Helmholtz resonators in the sample, absorbing 100 Hz noise wavelengths is challenging unless each resonator is sufficiently thick. Consequently, a double-layer design was implemented to improve the absorption of 100 Hz noise.

### Structural Design and Theoretical Mode

**Figure 1:**
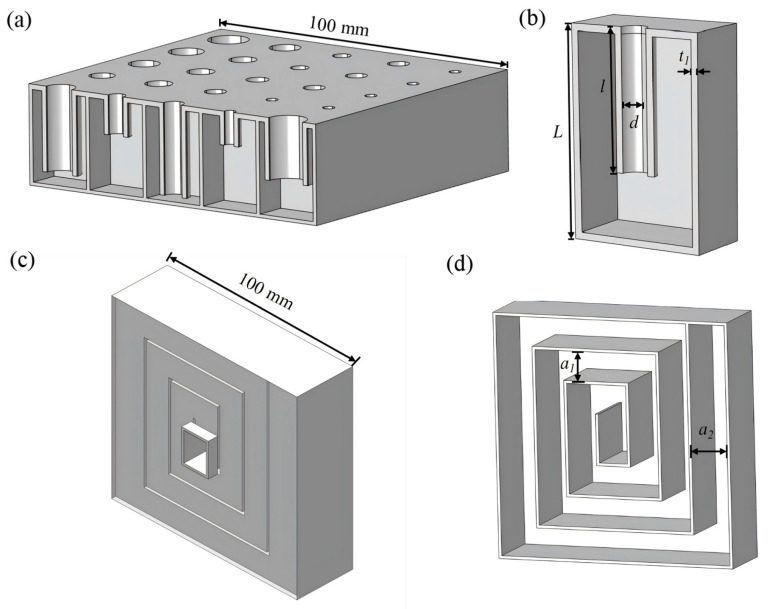
Structure of the metamaterial for sound absorption: (**a**) Upper component with parallel-connected Helmholtz resonators, with part removed for better visibility of the internal arrangement; (**b**) Half of the component removed to display the internal structure of a single Helmholtz resonator; (**c**) Lower component, featuring a Helmholtz resonator with a helical cavity, with part removed to show the internal structure; (**d**) Partially removed helical channel.

The schematic diagram of the proposed metamaterial is illustrated in Figure 1. The material consists of a two-layer composite structure: the upper component comprises a series of Helmholtz resonators connected in parallel (Figure 1a), and the lower component features a Helmholtz resonator with a helical cavity (Figure 1c). These components are linked by grooves, with the length of the resonator neck tube matching that of the helical cavity. The sample dimensions are 100 mm in length, 100 mm in width, and 60 mm in height. Key parameters include l and d, representing the length and diameter of the embedded tube, respectively; t1, the material thickness; and a1 and a2, the side lengths of the helical cavity.

The acoustic absorption performance of the Helmholtz resonator is primarily determined by its structural parameters [43]. To achieve broadband acoustic absorption, individual cavities are connected in parallel, providing high absorption efficiency and a broad low-frequency bandwidth, while maintaining a simple structure [44,45]. These Helmholtz resonators are specifically designed to target key noise frequencies observed in transformer equipment, as detailed in Table 1. They are optimized to absorb low-frequency noise, primarily in the 100 Hz range, which is important for addressing core noise and vibrations in transformers.

HR arrays have been widely investigated for low-frequency sound absorption, yet conventional configurations typically require compromises among absorption bandwidth, low-frequency effectiveness, and structural compactness. To overcome these constraints, we propose a two-layer hybrid structure that combines a helical cavity as the base layer with an array of parallel-connected HRs as the upper layer.

The helical cavity is designed to create an extended acoustic path within a compact footprint, enabling strong, frequency-selective attenuation of difficult low-frequency tonal components such as the 100 Hz transformer peak. Achieving comparable absorption at this frequency with a traditional HR would generally require a substantially thicker structure. In contrast, the upper HR array is engineered to deliver efficient absorption across the mid- to high-frequency range; its geometric parameters are jointly optimized with those of the helical base using a genetic algorithm to ensure complementary performance. This coordinated, stratified approach is essential: rather than simply stacking two independent absorbers, the design forms a coupled system in which the helical cavity suppresses the dominant low-frequency peak while the optimized HR array broadens the overall absorption spectrum. Consequently, the proposed structure achieves simultaneous targeted suppression at very low frequencies and broadband absorption while maintaining a limited total thickness. This yields a response tailored to substation noise characteristics and improves spatial efficiency and frequency-specific performance relative to comparably sized Helmholtz-based metamaterials.

In addition to addressing low-frequency noise, the metamaterial is designed for broadband noise reduction, which is particularly relevant for transformer cooling fans. This involves targeting frequencies between 500 Hz and 1300 Hz, which are associated with higher-frequency components of the noise. The selection of these frequencies is based on the peak noise frequencies observed in various transformer types and the necessity to cover both tonal and broadband components of transformer-generated noise.

Moreover, the design of each resonator allows for the effective adjustment of the absorption peak and frequency of the metamaterial. Given that substation noise generally corresponds to relatively low frequencies, requiring larger wavelengths, a genetic algorithm is employed to optimize the low-frequency sound absorption model. Each resonator is treated as a discrete variable, with the acoustic absorption coefficient serving as the objective function. By optimizing the structural parameters of each resonator, the overall structure achieves effective absorption within the target frequency range. Specifically, the Helmholtz resonator with a helical cavity provides excellent absorption at 100 Hz, while the parallel-connected Helmholtz resonators ensure broad frequency range absorption.

In the proposed two-layer hybrid structure, the lower helical cavity is selectively optimized by extending the acoustic propagation path to suppress the critical low-frequency peak at 100 Hz, as shown in Figure 1c,d. The upper Helmholtz resonator (HR) array is designed to provide broadband absorption in the mid- to high-frequency range, as shown in Figure 1a,b. The array comprises 25 HR unit cells, with geometric parameters summarized in Table 2. Each unit exhibits a distinct characteristic frequency, and together the units span the dominant frequency band of substation noise. Specifically, a subset of resonators is tuned to the additional low-frequency component at 300 Hz, whereas the remaining resonators are coordinated to cover broadband noise from 500 to 1400 Hz. This stratified, frequency-targeted architecture enables efficient absorption across the full substation noise spectrum.

The acoustic absorption coefficient α of the metamaterials is related to their acoustic impedance, which can be expressed as follows:

(1) $$\alpha =1-{\left|\frac{Z_{s}-1}{Z_{s}+1}\right|}^{2}$$

where *Z_s_* represents the surface acoustic impedance of the Helmholtz resonator.

The resonator includes an embedded neck, and the impedance of the inserted tube, *Z_n_*, is given by:

(2) $$Z_{n}=jw\rho_{0}l\left\{\frac{v_{0}}{jwq_{0}}\left\{1-\chi +\chi \sqrt{1+{\left(\frac{8\alpha_{\infty }q_{0}}{3\Lambda }\right)}^{2}}\frac{jw}{v_{0}}\right\}+\alpha_{\infty }\right\}$$

where *j* is the imaginary unit, *w* is the angular frequency, *f* is the sound wave frequency, $\rho_{0}$ is the air density, $v_{0\;}=\mu_{0}/\rho_{0}$ is the air viscosity $\mu_{0}=1.81\times 10^{-5}Pa\cdot s$ is the kinematic viscosity of air, $\sigma =32\mu_{0}/d^{2}$ is the static flow resistivity, $\Lambda =\sqrt{8\mu_{0}\alpha_{\infty }/\sigma }$ is the characteristic length of viscosity, $q_{0}=\mu_{0}/\sigma$ is the viscous permeability, $\alpha_{\infty }=1$ is the curvature, χ=1 is the curvature coefficient. To adjust the eigen-impedance $Z_{n}^{\prime }$ of the tube’s end, the following expression is used:

(3) $$Z_{n}^{\prime }=Z_{n}+\frac{4\sqrt{2}\mu_{0}y}{d}+0.85djw\rho_{0}$$

where $y=d\sqrt{\rho_{0}w/4\mu_{0}}$ is a dimensionless constant that represents the ratio of the neck radius to the thickness of the viscous boundary. The cavity characteristic impedance $Z_{c}$ can be expressed by:

(4) $$Z_{c}=-jZ_{0}cot(k_{0}\delta_{1}h)$$

where $Z_{0}=\rho_{0}c_{0}$ is the air characteristic impedance, $c_{0}$ is sound speed, h is the height of the internal air cavity, $k_{0}$ is the air wave number, $\delta_{1}=\left(V_{c}-V_{e}\right)/V_{c}$, $V_{c}$ is the air volume of the cavity, $V_{e}$ is the air volume of the inserted tube, $V_{c}=S_{c}\left(L-2t_{1}\right)$, $V_{e}=S_{e}\left(l_{e}-t_{1}\right)$, *L* is the overall thickness of the structure, and *l*_e_ is the length of the embedded pipe including the wall thickness.

The surface acoustic impedance for a single Helmholtz resonator is given by:

(5) $$Z_{s}=\delta_{2}(Z_{n}^{\prime }/\phi +Z_{c})/Z_{0}$$

where $\delta_{2}$ is the correction factor considering the effect of cavity thickness on surface acoustic impedance, $\delta_{2}=S_{A}/S_{c}$, $S_{A}$ is the cross-sectional area of the external cavity, $S_{c}$ is the cross-sectional area of the internal air cavity, ϕ is the perforation rate, $\phi =S_{e}/S_{c}$, and $S_{e}$ is the cross-sectional area of the inserted tube. After parallel connection, the total surface impedance $Z_{u}$ can be determined by:

(6) $$Z_{u}=n\delta \cdot {\left(\sum_{k=1}^{k=n}\frac{1}{Z_{k}}\right)}^{-1}$$

where *n* is the number of cavities, and $Z_{k}$ is the surface acoustic impedance of a single resonator.

## 3. Simulation Results and Discussion

In the design process, structural parameters must be optimized to achieve the best acoustic absorption coefficient at a specific frequency [46,47,48]. Once the overall dimensions of the model are determined, the acoustic absorption performance is fine-tuned by adjusting the cavity opening diameter d and neck depth l. The genetic algorithm was employed to optimize the structural parameters of the Helmholtz resonators to achieve effective absorption across a broad frequency range. For the low-frequency design, a specific focus was placed on achieving high absorption at 100 Hz to mitigate core noise. Additionally, to address the broadband noise observed between 500 Hz and 1300 Hz, the design was further optimized for these frequencies, ensuring the metamaterial’s ability to reduce noise effectively across both low- and high-frequency ranges. The parameters of the resonators were chosen based on their ability to match the peak frequencies identified in Table 1, ensuring that the metamaterial would be effective for a broad range of transformer-generated noises. The crossover probability is set to 80%, the mutation probability is set to 5%, and the fitness function is set according to Equations (1)–(5). The objective function is defined as the acoustic absorption coefficient at the specified frequency, with values closer to 1 indicating higher absorption. This implies that the corresponding parameter combination is more likely to be selected and retained. Initially, a broadband acoustic absorption material sample was designed and experimentally validated through simulations and an impedance tube, ensuring the accuracy of the optimization algorithm. The algorithm was then applied to design low-frequency acoustic absorption materials.

**Figure 2:**
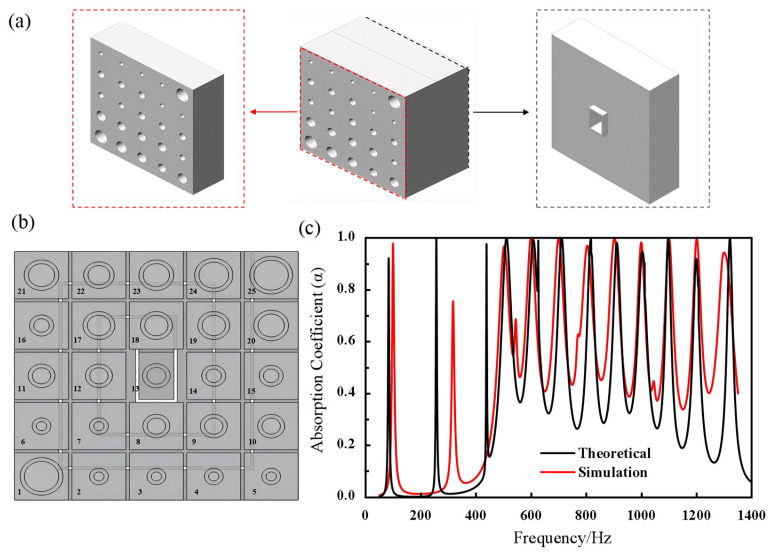
(**a**) Schematic of the overall structure of the acoustic metamaterial, including the upper and lower components. (**b**) Numbering of the components in the acoustic metamaterial. (**c**) Comparison of the acoustic absorption coefficients between the theoretical calculations and simulation results of the acoustic metamaterial.

The final low-frequency design comprises 25 Helmholtz resonators (HRs). The structural diagram of the 3D acoustic metamaterial is shown in Figure 2a, with the red dashed box highlighting the upper component and the black dashed box highlighting the lower component. In Figure 2b, the 25 resonators are numbered for reference. The parameters for the neck diameter and length of the parallel Helmholtz resonators, optimized using a genetic algorithm, along with the width and spiral length of the Helmholtz resonators with coiled channels, are listed in Table 2 and Table 3. “NO.” refers to the serial number of an individual Helmholtz resonator. As shown in Figure 1a, the upper layer consists of 25 Helmholtz resonators arranged in parallel. This arrangement is also shown in the cross-sectional view of Figure 2b, where each resonator is labeled with a number from 1 to 25.

To evaluate the acoustic absorption performance of the Helmholtz resonators, simulations were conducted using COMSOL Multiphysics version 6.3 with the Pressure Acoustics physics interface. A plane wave incident condition was applied to model the acoustic excitation, and the thermo-viscous boundary layer impedance was included to account for viscosity and thermal conduction effects at the material interface. The model’s accuracy in predicting the material’s performance across the specified frequency range was validated by comparison with experimental data.

**Figure 3:**
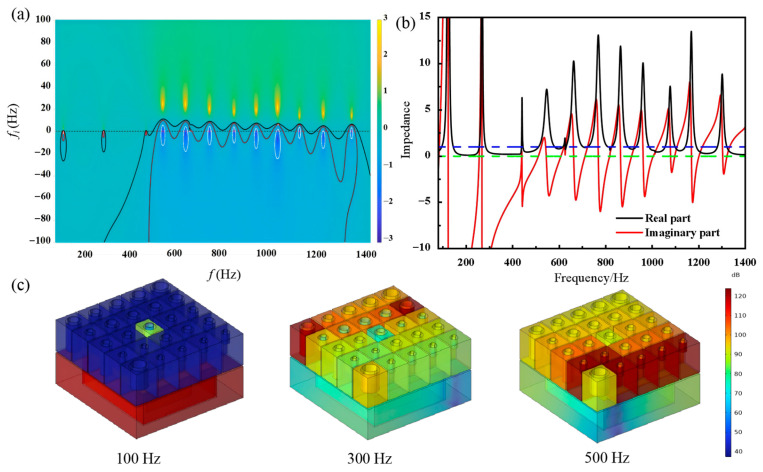
Acoustic Analysis of the Absorptive Metamaterial (**a**) Complex Frequency Plane diagram; (**b**) Impedance Matching diagram; (**c**) Sound Pressure Level Distribution at 100 Hz, 300 Hz, and 500 Hz.

Subsequently, the acoustic absorption performance of the proposed structure at the target frequency was analyzed using a complex frequency plane plot (Figure 3a). In this plot, the x-axis represents the real frequency, and the y-axis represents the imaginary frequency. The complex reflection coefficients for acoustic absorption values of 0.25, 0.5, and 0.9 are depicted by black, red, and white lines, respectively. Zero points are indicated by blue dots, while yellow dots represent pole. These points appear in pairs and are symmetrically distributed. At complete acoustic absorption, the zero points align with the real frequency axis. However, due to inherent losses in the acoustic system, both the zero and pole points shift toward the positive direction of the imaginary frequency axis. The zero and pole points were observed at frequencies of 100 Hz, 300 Hz, 500 Hz, 600 Hz, 700 Hz, 800 Hz, 900 Hz, 1000 Hz, 1100 Hz, 1200 Hz, and 1300 Hz. The proximity of the zero points to the real frequency axis indicates strong acoustic absorption performance at these frequencies.

Further analysis of the structure’s acoustic absorption performance was conducted using impedance matching theory (see Figure 3b). As illustrated in this figure, the blue dashed line represents a value of 1, while the green dashed line represents 0. At the target frequencies, the real part of the normalized acoustic impedance approaches 1, and the imaginary part approaches 0, satisfying the ideal impedance-matching condition and resulting in near-perfect acoustic energy dissipation.

Unlike the application of electrochemical impedance spectroscopy (EIS) to analyze charge dynamics at electrolyte interfaces [49], acoustic impedance measures the ease or difficulty with which sound waves propagate through specific media, thus characterizing the intrinsic physical properties of materials. A greater mismatch in acoustic impedance results in more energy being reflected at the boundary between two media. While this approach is applied here to sound absorption, it shares a common mathematical framework with techniques like EIS. Both rely on interpreting the system’s complex response (Z(ω) = Z′(ω) + jZ″(ω)) through physical or equivalent-circuit models to analyze loss mechanisms and resonance behaviors.

In this context, the genetic algorithm’s optimization of geometric parameters can be viewed as tuning the components of an equivalent acoustic circuit. The algorithm systematically adjusts these parameters to minimize impedance mismatch with air across the target frequency band, directly linking the structural design to the desired performance outcome—maximized broadband absorption. The frequency points in Figure 3b, where the normalized real part is close to 1 and the imaginary part is close to 0, correspond to perfect impedance matching and maximum acoustic energy dissipation, resulting in an absorption coefficient αapproaching 1. Each extremum in the impedance curve corresponds to a specific resonant mode within the metamaterial. At frequencies of 100 Hz, 300 Hz, 500 Hz, 600 Hz, 700 Hz, 800 Hz, 900 Hz, 1000 Hz, 1100 Hz, 1200 Hz, and 1300 Hz, the real part of the impedance is close to 1, and the imaginary part is close to 0. This suggests good acoustic impedance matching and excellent acoustic absorption performance at these frequencies.

Figure 3c shows that the lower helical cavity structure generates high sound pressure levels through resonance, effectively absorbing noise at 100 Hz. However, due to its large size, it is not suitable for designing multiple low-frequency absorbers. In the upper acoustic structure, different Helmholtz resonators absorb noise at various frequencies, achieving broadband sound absorption. As shown in Figure 3c, strong resonance occurs at the resonant frequencies of 100 Hz, 300 Hz, and 500 Hz, contributing to effective noise absorption.

## 4. Experimental Validation

**Figure 4:**
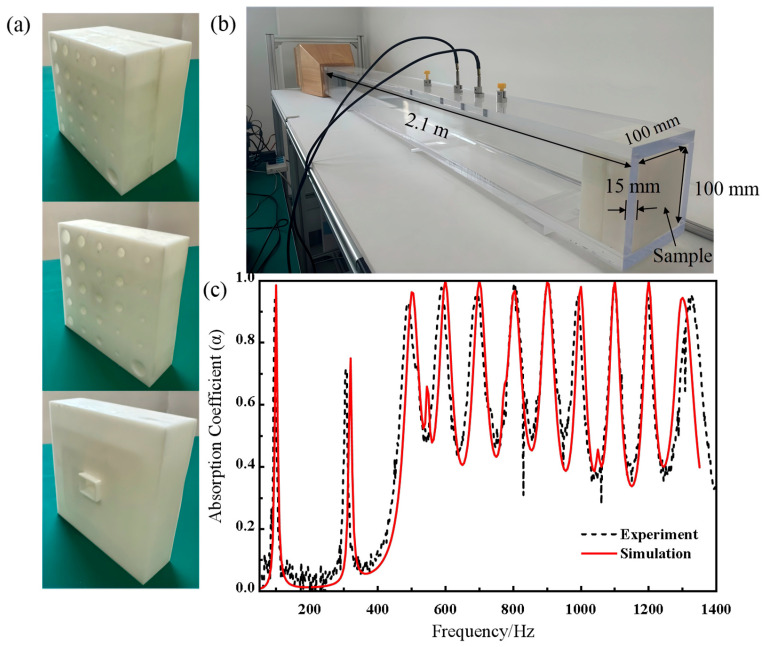
Acoustic experiment of the broadband sound-absorbing metamaterial: (**a**) 3D-printed sample, (**b**) experimental setup, (**c**) experimental and simulation sound absorption coefficients.

To evaluate the sound absorption performance of the metamaterial, we fabricated samples using 3D printing technology. Figure 4a illustrates the overall structure, including both the upper and lower components of the sample. The design was fabricated using stereolithography (SLA) 3D printing (lite800, UnionTech, Inc., Shanghai, China) with a photosensitive resin material. The experimental setup is shown in Figure 4b, which utilizes an impedance tube (100 mm × 100 mm) and the two-microphone method for testing. The sample is placed at the end of the impedance tube for measurement. Experimental data and simulation results are shown in Figure 4c. The results indicate that the proposed metamaterial exhibits excellent sound absorption performance at the target frequencies, with absorption coefficients exceeding 0.9. The experimental data closely align with the simulation results, although minor discrepancies at the peak frequencies may be due to the precision limitations inherent in the 3D printing process. Overall, the findings confirm that the metamaterial effectively reduces noise in substations.

## 5. Conclusions

This study presents the design and experimental validation of a broadband sound-absorbing metamaterial tailored for low-frequency noise control in substations. The metamaterial is engineered to effectively absorb sound at key frequency ranges, including 100 Hz, 300 Hz, and 500 Hz to 1300 Hz. These ranges are critical for mitigating both the core noise emitted by transformers at 100 Hz and 300 Hz and the broadband noise produced by transformer cooling systems in the 500 Hz–1300 Hz range. Structural optimization using a genetic algorithm ensures the metamaterial performs efficiently across these frequencies, providing targeted absorption where it is most needed. The outcome is a highly effective noise control solution that addresses both low-frequency and broadband noise in substations, improving the overall acoustic environment and offering reliable noise reduction across a wide range of transformer-related frequencies. This study highlights the material’s practical application and its potential for broader use in noise-sensitive environments.

## Figures and Tables

**Table 1:** Spectral characteristics of transformer noises.

Equipment	Peak Noise Frequencies/Hz
10 kV distribution transformer	50, 100, 200, 300
35 kV distribution transformer	50, 100, 300, 400
110 kV transformer	50, 100, 300
220 kV transformer	50, 100, 200, 300, 400, 500
500 kV transformer	100, 500, 800

**Table 2:** Parameters of upper acoustic absorption structure.

NO.	Opening Diameter of Each Component *d*/mm	Neck Depth of Each Component *l*/mm
1	11.8	18
2~7	3.5	9.7
8~12	6.3	26.5
13	6.6	8.2
14~16	5.3	11.4
17~19	8	24.3
20~21	9	24.6
22	7.5	9.9
23	9	12.9
24	10.2	14.3
25	11.8	18

**Table 3:** Parameters of lower acoustic absorption structure.

NO.	Width of Each Component *d*/mm	Length of Each Component *l*/mm
*a*_1_	12	622.6
*a*_2_	15.2	181.6

## Data Availability

The original contributions presented in this study are included in the article. Further inquiries can be directed to the corresponding authors.
